# Duloxetine for painful diabetic neuropathy and fibromyalgia pain: systematic review of randomised trials

**DOI:** 10.1186/1471-2377-8-29

**Published:** 2008-08-01

**Authors:** Asquad Sultan, Helen Gaskell, Sheena Derry, R Andrew Moore

**Affiliations:** 1 Nuffield Department of Anaesthetics, John Radcliffe Hospital, Oxford, OX3 9DU, UK; 2 Pain Research, Churchill Hospital, Oxford, OX3 7LJ, UK

## Abstract

**Background:**

Duloxetine hydrochloride is a reuptake inhibitor of 5-hydroxytryptamine and norepinephrine used to treat depression, generalized anxiety disorder, neuropathic pain, and stress incontinence in women. We investigated the efficacy of duloxetine in painful diabetic neuropathy and fibromyalgia to allow comparison with other antidepressants.

**Methods:**

We searched PubMed, EMBASE (via Ovid), and Cochrane CENTRAL up to June 2008 for randomised controlled trials using duloxetine to treat neuropathic pain.

**Results:**

We identified six trials with 1,696 patients: 1,510 were treated with duloxetine and 706 with placebo. All patients had established baseline pain of at least moderate severity. Trial duration was 12 to 13 weeks. Three trials enrolled patients with painful diabetic neuropathy (PDN) and three enrolled patients with fibromyalgia. The number needed to treat (NNT) for at least 50% pain relief at 12 to 13 weeks with duloxetine 60 mg versus placebo (1,211 patients in the total comparison) was 5.8 (95% CI 4.5 to 8.4), and for duloxetine 120 mg (1,410 patients) was 5.7 (4.5 to 5.7). There was no difference in NNTs between PDN and fibromyalgia. With all doses of duloxetine combined (20/60/120 mg) there were fewer withdrawals for lack of efficacy than with placebo (number needed to treat to prevent one withdrawal 20 (13 to 42)), but more withdrawals due to adverse events (number needed to harm (NNH) 15 (11 to 25)). Nausea, somnolence, constipation, and reduced appetite were all more common with duloxetine than placebo (NNH values 6.3, 11, 11, and 18 respectively). The results for duloxetine are compared with published data for other antidepressants in neuropathic pain.

**Conclusion:**

Duloxetine is equally effective for the treatment of PDN and fibromyalgia, judged by the outcome of at least 50% pain relief over 12 weeks, and is well tolerated. The NNT of 6 for 50% pain relief suggests that this is likely to be a useful drug in these difficult-to-treat conditions, where typically only a minority of patients respond. Comparing duloxetine with antidepressants for pain relief in DPN shows inadequacies in the evidence for efficacy of antidepressants, which are currently recommended in PDN care pathways.

## Background

Neuropathic pain is the consequence of damage to the central nervous system (e.g. cerebrovascular accident, multiple sclerosis or spinal cord injury) or peripheral nervous system (e.g. painful diabetic neuropathy (PDN), postherpetic neuralgia (PHN), surgery). It has a significant negative impact on quality of life [1]. Some patients with neuropathic pain respond well to treatment and others show no obvious response [2-4]. No pharmacological intervention produces meaningful relief for more than half the patients with neuropathic pain [5].

The incidence of PHN and trigeminal neuralgia and PDN together is almost 0.1% per year in the UK [6]. The incidence of neuropathic pain is growing, presumably because of increased numbers of older persons and diabetics, amongst whom about one in five develop painful neuropathy at some stage. Neuropathic pain is quite common in general medical practice with about 1% point prevalence in UK if fibromyalgia, PDN, PHN, and trigeminal neuralgia are included [7].

The most common pharmacological approaches to the management of neuropathic pain include antidepressants (tricyclic antidepressants, serotonin and norepinephrine reuptake inhibitors), antiepileptics (valproate, carbamazepine, gabapentin, pregabalin), opioids, other analgesics, topical lidocaine patch, and topical capsaicin. The evidence for these has been reviewed extensively [4,8-16].

5-hydroxytryptamine (5HT) and norepinephrine (NE) are involved in the modulation of endogenous analgesic mechanisms via descending inhibitory pain pathways in the brain and spinal cord [17]. Disinhibition and imbalance of 5HT and NE in endogenous pain inhibitory pathways could contribute to persistent pain. An increase in 5HT and NE may increase inhibition of painful signals, improving pain relief.

Duloxetine hydrochloride is a serotonin-norepinephrine reuptake inhibitor used to treat depression, generalized anxiety disorder, neuropathic pain, and stress incontinence in women. We investigated the efficacy of duloxetine in the management of PDN and fibromyalgia as duloxetine had not been included in the most recent systematic reviews, including one of antidepressants [13]. Duloxetine in PDN alone has been the subject of a recent post hoc analysis [18].

## Methods

We searched PubMed, EMBASE (via Ovid), and Cochrane CENTRAL up to June 2008 for randomised controlled trials using duloxetine to treat neuropathic pain. The detailed search strategy included use of the drug name "duloxetine" anywhere in an article, together with "randomized controlled trial" as subject heading, publication type or text word; this was modified appropriately for different databases. Reference lists of retrieved articles and reviews were also searched for relevant trials. We contacted Boehringer Ingelheim Limited as a UK distributor for duloxetine in neuropathic pain to enquire about relevant published or unpublished studies, and examined an on-line register [19].

Included trials had to be randomised, double blind, placebo controlled, and use duloxetine to treat adult patients with painful neuropathies of any cause. Trials had to have a minimum of 10 patients per treatment arm, and a planned duration of at least four weeks.

The abstracts were read, and potentially useful reports retrieved in full. No information was taken from posters or abstracts. Decisions on inclusion or exclusion of trials, assessment of trial quality and validity and all data extraction were made independently by three reviewers, with discrepancies resolved by consensus.

Methodological quality of included studies was assessed using a validated 5-point scale [20] utilising reporting of randomisation, blinding, and withdrawals. The maximum score possible was 5 points, and no study could be included with fewer than 2 points (one for randomisation and one for blinding). Study validity was assessed using a validated 16-point scale [21].

Data were abstracted into a standard form. Information extracted from the trials included details of the patients (number, age, sex, pain syndrome), duloxetine dose, and permitted rescue analgesia. The primary outcome sought was 50% pain relief. Other measures of pain relief were abstracted where reported. Secondary outcomes were withdrawals (all cause, lack of efficacy and adverse events) and adverse events (patients with at least one adverse event, serious adverse events, and specific adverse events).

Guidelines for quality of reporting of meta-analyses were followed where appropriate [22]. The prior intention was to pool data where there was clinical and methodological homogeneity, with similar patients, dose, duration, outcomes, and comparators, but not where numbers of events were small, and random chance might well dominate effects of treatment [23]. Homogeneity tests and funnel plots, though commonly used in meta-analysis, were not used because they have been found to be unreliable [24,25]. Instead, clinical homogeneity was examined graphically [26]. Relative benefit (or risk) and number needed to treat or harm (NNT or NNH) were calculated with 95% confidence intervals. Relative benefit or risk was calculated using a fixed effects model [27] with no statistically significant difference between treatments assumed when the 95% confidence intervals included unity. We added 0.5 to treatment and comparator arms of trials in which at least one arm had no events. NNT or NNH was calculated [28] using the pooled number of observations only when there was a statistically significant difference of relative benefit or risk (where the confidence interval did not include 1). We used the following definitions:

• When significantly more beneficial outcomes occurred with duloxetine than placebo, we used the term number needed to treat (NNT).

• When significantly fewer adverse events occurred with duloxetine than placebo we used the term the number-needed-to-treat to prevent one adverse event (NNTp).

• When significantly more adverse events occurred with duloxetine than placebo we used the term the number-needed-to-harm to cause one adverse event (NNH).

Statistical significance of any difference between NNT for different doses was assumed if there was no overlap of the confidence intervals, and additionally tested using the z statistic [29]. RevMan 5.0.12 was used to analyse continuous data. There was a prior intention to carry out sensitivity analyses for high versus low trial quality (<3 vs ≥ 3) and validity (<9 vs ≥ 9), duloxetine dose, and pain syndrome. A minimum of two trials and 250 patients was required in any sensitivity analysis [23].

## Results

We identified six trials satisfying the inclusion criteria [30-35]. Details of the included studies are in Additional File 1. A total of 2,216 patients were included, 1,510 treated with duloxetine and 706 with placebo. Three trials [32-34] enrolled patients with PDN and three [30,31,35] enrolled patients with fibromyalgia, in which 23% to 38% had a diagnosis of major depressive disorder. The trials in PDN excluded patients with any diagnosed psychological disorder. We did not include any trials in which the primary problem was a major psychiatric disorder but with a secondary painful condition [36-41]. All patients had established baseline pain of at least moderate severity, measured using established scales. The mean age in the trials ranged between 49 and 61 years, and the majority of patients were Caucasian. One trial [31] enrolled only women, and the others between 5% and 61% men.

Trial duration was 12 to 13 weeks. One trial [33] had a 13-week continuation phase, but results for the first 13 weeks (acute phase) only are analysed here, to make it comparable with the other trials. Duloxetine was used at doses of 20, 60, or 120 mg daily, with titration up to the 120 mg dose, which was given as a divided dose of 60 mg twice daily. Up to 2 g acetaminophen daily was permitted as rescue medication in the fibromyalgia trials, and up to 4 g daily in the PDN trials.

Trials were of good methodological quality, with three scoring 5/5, two scoring 4/5, and one scoring 3/5 on the Oxford Quality Score [20]. Two scored 16/16 and four scored 13/16 on the Oxford Pain Validity Score [21]. No sensitivity analyses were therefore carried out for these criteria.

### Efficacy

#### 50% Pain Relief

**Figure 1:**
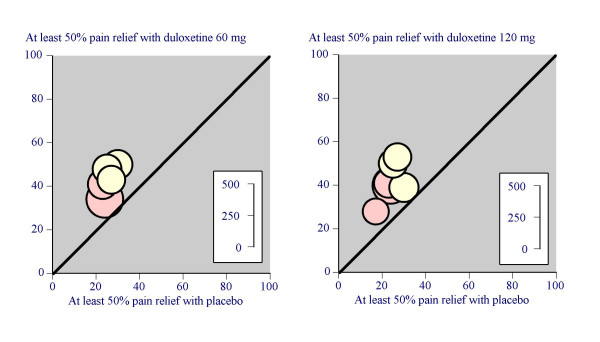
Proportion of patients with at least 50% pain relief with duloxetine 60 mg or 120 mg and placebo in individual trials. Pink circles are fibromyalgia trials. Inset scale shows number in comparison.

All six trials reported the outcome of at least 50% pain relief over baseline in the 24-hour average pain score by the end of the trial, and results are summarised in Figure 1 and Table 1. Trials were consistent, and overall 41% of patients achieved 50% pain relief with any dose of duloxetine compared with 24% with placebo. Combining all doses in both conditions (2,216 patients), the NNT for one patient to achieve at least 50% pain relief with duloxetine compared with placebo was 5.9 (95% CI 4.8 to 7.7).

**Table 1:** Summary of efficacy and adverse event outcomes in duloxetine trials

		**Number of**		**Percent with**			
				
**Outcome**	**Dose (daily maximum)**	**Trials**	**Patients**	**Duloxetine**	**Placebo**	**Relative benefit or risk (95% CI)**	**NNT/NNTp/*NNH *(95% CI)**
**Efficacy**							
50% PR All	20/60/120 mg	6	2,216	41	24	1.7 (1.4 to 1.9)	**5.9 (4.8 to 7.7)**
50% PR PDN	60/120 mg	3	1,024	47	27	1.7 (1.4 to 2.1)	**5.1 (3.9 to 7.3)**
50% PR fibromyalgia	60/120 mg	3	996	37	21	1.7 (1.4 to 2.1)	**6.4 (4.7 to 9.9)**
50% PR	60 mg	5	1,211	43	26	1.7 (1.4 to 2.0)	**5.8 (4.4 to 8.4)**
50% PR	120 mg	6	1,410	42	24	1.7 (1.5 to 2.0)	**5.7 (4.5 to 7.8)**
**Adverse events general**							
Withdrawal – all cause	20/60/120 mg	6	2,418	30	26	1.2 (1.1 to 1.4)	*26 (13 to 426)*
Withdrawal – LoE	20/60/120 mg	5	1,872	4	9	0.5 (0.4 to 0.7)	20 (13 to 42)
Withdrawal – AE	20/60/120 mg	6	2,220	15	8	1.8 (1.4 to 2.4)	*15 (11 to 25)*
Any AE	60/120 mg	4	1,243	82	67	1.2 (1.2 to 1.3)	*6.7 (5.0 to 10)*
Serious AE	60/120 mg	3	1,034	2	3	0.8 (0.4 to 1.7)	not calculated
**Specific adverse events**							
Nausea	20/60/120 mg	3	1,145	29	10	3.0 (2.2 to 4.3)	*5.3 (4.3 to 6.9)*
Somnolence	20/60/120 mg	3	1,145	14	4	2.9 (1.7 to 4.9)	*11 (8.0 to 16)*
Constipation	20/60/120 mg	3	1,145	13	3	3.6 (2.0 to 6.5)	*11 (8.3 to 16)*
Decreased appetite	20/60/120 mg	2	811	7	1	4.9 (1.7 to 14)	*18 (12 to 34)*

PR – pain relief; PDN – painful diabetic neuropathy; LoE – lack of efficacy; AE – adverse event

In the right hand column, bold font is used for **NNT – number needed to treat**; normal font for NNTp – number need to treat to prevent; italic font for *NNH – number needed to harm*

Five of the trials used 60 mg, and all six used 120 mg; only 66 patients (in two treatment arms) received the 20 mg dose. The dose of duloxetine made little difference to the result (Figure 1, Table 1). There was no difference in the proportion of patients achieving at least 50% pain relief with 60 mg and 120 mg (z = 0.13; p = 0.89).

There was no significant difference in the proportion of patients achieving at least 50% pain relief with PDN or fibromyalgia (z = 0.95; p = 0.34). The proportion of patients with this outcome was slightly lower for both placebo and duloxetine groups in the fibromyalgia trials (Table 1), with similar NNTs for both conditions.

#### Average pain score (APS)

**Figure 2:**
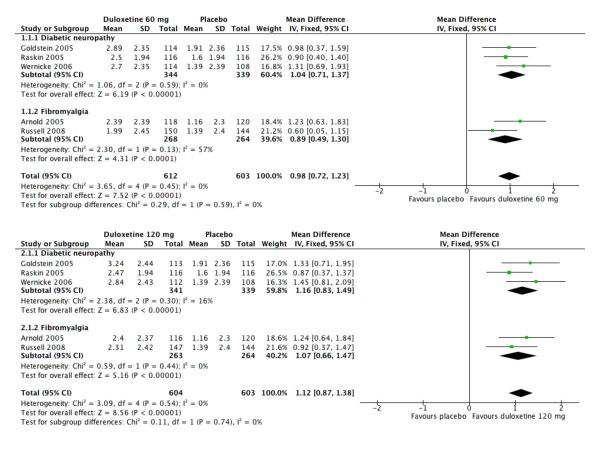
Mean change from baseline to endpoint on the 24-hour average pain score (APS) for treatment compared to placebo over 12 to 13 weeks, by duloxetine dose (60 mg and 120 mg) and condition (diabetic neuropathy and fibromyalgia).

Five of the trials [31-35] recorded daily 24-hour average pain scores (APS) on a 0–10 scale, and reported this as a weekly mean, as well as the change from baseline to final weekly mean. The change in weekly mean APS on treatment was compared with placebo over the 12 or 13 weeks. Figure 2 shows the calculations for different doses of duloxetine in different pain syndromes. The weighted mean difference for duloxetine 60 mg compared with placebo was 1.0 (0.71 to 1.4), and for duloxetine 120 mg compared with placebo was 0.9 (0.49 to 1.3). There was no difference in response between patients with PDN and fibromyalgia.

#### Withdrawals

Withdrawals for any cause occurred in slightly more patients with duloxetine (30%) than placebo (28%); the NNTp for all cause withdrawal with duloxetine rather than placebo was 26 (13 to 426) (Table 1). Withdrawals due to lack of efficacy occurred in significantly fewer patients (4%) taking duloxetine than placebo (9%); the NNTp for lack of efficacy withdrawal with duloxetine rather than placebo was 17 (12 to 35) (Table 1).

Withdrawals for any cause or for lack of efficacy did not differ significantly between the 60 mg and 120 mg doses, although for any cause they were consistently 4% to 5% lower for 60 mg than 120 mg, except for Russell et al [35] where the rates were almost identical.

### Adverse events

#### Withdrawals

Withdrawals due to adverse events occurred significantly more often with duloxetine (15%) than placebo (8%). The NNH was 15 (11 to 25) (Table 1). They were 2% to 8% lower with 60 mg than 120 mg, giving an NNH of 19 (11 to 86) for 120 mg compared to 60 mg.

#### Any adverse event

The "at least one adverse event" criterion was met in significantly more patients taking duloxetine (82%) than placebo (67%) in the four trials that reported this outcome. The NNH was 6.7 (5.0 to 10) (Table 1).

#### Serious adverse events

Serious adverse events were reported in only three trials; one trial did not report this outcome [30], one did not report it for the 13-week phase [35], and one did not separate rates between groups [32]. In the three trials reporting serious adverse events they were uncommon and not significantly different between duloxetine or placebo, at about 2–3% over the 12 weeks of the trials (Table 1). Russell et al [35] reported that serious adverse events were infrequent over the full 6 months of the trial.

#### Specific adverse events

Only three trials [31,32,34] provided numbers of patients experiencing specific treatment emergent adverse events over 12 to 13 weeks. There were statistically significant increases in nausea (29% vs 10%), somnolence (14% vs 4%), constipation (13% vs 3%) and decreased appetite (7% vs 1%) with all doses of duloxetine compared with placebo (Table 1). There were small mean increases in laboratory tests and vital signs, but these were transient and not considered clinically relevant by the trialists.

## Discussion

This systematic review differs from the only other that considers duloxetine [18]. That company-sponsored review was able to pool data from the three PDN trials. It calculated NNTs for at least 50% pain relief (with identical results to those calculated here), and also gave NNTs for at least 30% pain relief. It demonstrated the stability of NNTs over two to 12 weeks, an important observation, and no difference in estimate depending on treatment of dropouts. This review differs in demonstrating that the efficacy of duloxetine is similar in PDN and fibromyalgia, and also makes an informed comparison with other evidence on antidepressant treatments for neuropathic pain.

For evidence to be credible, it has to fulfil criteria of quality, validity, and size [42]. The evidence here on duloxetine does that. Trials were randomised, and double blind, and quality and validity scores indicated low chance of bias. The trials were of sufficient length (12 or 13 weeks) to make them clinically relevant, and the outcome reported of at least 50% pain relief was a high hurdle. Most older neuropathic pain studies used less stringent measures, including undefined "improvement" as an outcome, and only trials of pregabalin have also consistently used at least 50% pain relief. Finally, with information on over 2,200 patients, including over 1,000 patients with PDN, the data set for duloxetine fulfils the requirements of size [23] and is much larger than any previous data set for antidepressants in neuropathic pain [13].

Significantly more patients achieved the outcome of at least 50% pain relief with duloxetine (41%) than with placebo (24%) over 12 weeks. The outcome of 50% pain represents substantial clinical pain relief, and an NNT of 6 suggests that this is likely to be a useful drug in these difficult-to-treat conditions, where typically only a minority of patients respond. There was no dose response between 60 mg and 120 mg, nor was there any significant difference in the duloxetine response between PDN or fibromyalgia. There was a similar lack of dose response in this range for use of duloxetine in major depressive disorder [43].

Duloxetine was well tolerated in the trials, with fewer withdrawals due to adverse events with 60 mg than with 120 mg. Most adverse events were reported to be mild or moderate, with nausea, somnolence, constipation, decreased appetite and dry mouth frequently mentioned. In stress incontinence duloxetine affects the resting tone and contraction of the urethral striated sphincter muscle. It might be expected to cause symptoms of urinary hesitancy in patients without incontinence, but urinary problems were not reported in any of these trials, or in trials of duloxetine in depression [for example [44,45]].

This review has some limitations. Firstly, pain intensity measurements used to calculate our primary outcome of at least 50% pain relief were derived from average pain intensity scores during the previous 24 hours. Secondly, the trials were 12 to 13 weeks in duration, and although they demonstrated a sustained response and good tolerability over this period, they provided no information for longer-term efficacy or safety. Russell et al [35] included a 13-week continuation phase, and reported continuing efficacy and tolerability, as have open-label extension studies in neuropathic pain lasting 26 and 52 weeks [46,47].

Duloxetine has been widely trialled in other conditions, in particular depression and stress-induced incontinence in women, and the many trials have been subject to systematic review in those therapeutic areas. An evaluation of cardiovascular safety in 42 placebo controlled trials involving 8,500 patients concluded that duloxetine did not appear to be associated with significant cardiovascular risks [48].

Finally, the studies in fibromyalgia included some patients with depression. Although only a minority were depressed (Additional file 1), it could be argued that duloxetine reduced pain intensity by improving depression. We identified a small number of other trials in patients with psychiatric disorders with painful physical symptoms (not neuropathic pain) [36-41]. Although none reported our primary outcome of 50% pain relief, they did use the same scales to record pain intensity, and all reported improvements in pain with duloxetine 60 mg that did not entirely correlate with improvements in depression. Fava et al. estimated that 50% of the total effect of duloxetine on overall pain was independent of changes in depression [37]. A counter view was that duloxetine was ineffective in treating pain in depression [49]. In the trials included in this review, about one third of the patients with fibromyalgia also had major depressive disorder. It would be difficult to attribute all of the analgesic effect of duloxetine in these trials to improvements in depression in a minority, although one could not rule out a contributory effect. One of these trials estimated that around 20% of the overall treatment effect with 120 mg and 30% with 60 mg was due to improvements in depressive symptoms, the remainder being due to duloxetine's direct effect on pain reduction [35].

The quantity and quality of randomised trial data in neuropathic pain is limited. Table 2 shows the results for duloxetine 60 mg and 120 mg over 12 weeks compared with other results for antidepressants calculated from a recent Cochrane review [13]. Only patients with PDN are reported in Table 2 for duloxetine, in order to keep to similar inclusion criteria. Even so, the amount of evidence on duloxetine dominates the evidence available, almost doubling the number of patients studied previously with antidepressants. Table 2 shows the curious tendency for smaller amounts of information to be associated with greater benefit, either as higher values for relative risk or lower values for NNT. Size and quality may be linked: only one trial in the Cochrane meta-analysis had over 100 participants, while all the duloxetine trials had over 200, and many older studies used poorly defined outcomes of improvement, probably less stringent than that of at least 50% pain relief. Some small older studies also had a crossover rather than parallel design. This presents problems in determining relative efficacy among antidepressants for treatment of neuropathic pain.

**Table 2:** Summary of efficacy in antidepressant meta-analysis

		**Number of**		**Percent with**			
				
**Antidepressant**	**Outcome**	**Trials**	**Patients**	**Placebo**	**Active**	**Relative benefit (95% CI)**	**NNT (95% CI)**
Duloxetine 60/120 mg	at least 50% pain relief	3	1,024	27	47	1.7 (1.4 to 2.1)	5.1 (3.9 to 7.3)
Amitriptyline all doses	global improvement	10	588	32	64	2.0 (1.6 to 2.4)	3.2 (2.6 to 4.2)
Other antidepressants	global improvement	3	216	12	50	4.2 (2.5 to 7.0)	2.6 (2.0 to 3.7)
Venlafaxine all doses	global improvement	3	200	25	57	2.3 (1.6 to 3.4)	3.1 (2.2 to 5.1)
Desipramine all doses	global improvement	2	78	10	59	5.8 (2.2 to 15)	2.1 (1.5 to 3.3)
Imipramine all doses	global improvement	2	58	5	97	19 (3.9 to 89)	1.1 (1.0 to 1.2)

NNT – Number need to treat

Several evidence-based recommendations for the treatment of neuropathic pain place use of antidepressants early in any care pathway [5,9,50]. Comparing the evidence for different therapies within a class, and between classes, is key to determining the most effective, and most cost-effective pathway. In that circumstance, it is not enough just to calculate an NNT. The quality and credibility of the evidence behind those calculations needs to be evaluated, a function of the utility and validity of outcomes, and to have sufficient numbers of patients or events to avoid random chance. The example of duloxetine provides a firm evidential base for within and between class comparisons.

## Conclusion

Duloxetine is equally effective for the treatment of PDN and fibromyalgia, judged by the outcome of at least 50% pain relief over 12 to 13 weeks, and is well tolerated. The NNT of 6 for this outcome suggests that this is likely to be a useful drug in these difficult-to-treat conditions, where typically only a minority of patients respond. Doses higher than 60 mg do not provide additional pain relief, but do cause slightly more withdrawals due to adverse events. Comparing duloxetine with other antidepressants for pain relief in PDN shows inadequacies in the evidence for efficacy of antidepressants, which are currently recommended in PDN care pathways.

## Competing interests

RAM, and SD have received research support from charities, government and industry sources at various times, and HG from government, but no such support was received for this work. No author has any direct stock holding in any pharmaceutical company.

## Authors' contributions

AS, SD and HG carried out searches, selected studies and carried out data extraction. AS, SD and AM were involved with analysis. All authors were involved with writing, and all authors read and approved the final manuscript.

## Pre-publication history

The pre-publication history for this paper can be accessed here:



## Supplementary Material

Additional file 1 Included studies. Details of the design and outcomes of the included studies. Click here for file
