# Supplementary material for: Duloxetine for painful diabetic neuropathy and fibromyalgia pain: systematic review of randomised trials
Source: BMC Neurol. 2008 Aug 1;8:29. doi: 10.1186/1471-2377-8-29 (PMC2529342; doi:10.1186/1471-2377-8-29)
Supplement: Additional file 1 — Included studies. Details of the design and outcomes of the included studies. [file 1471-2377-8-29-S1.pdf]

Additional file 1: Included studies

Lead Author and Year	Condition/ Inclusion criteria	Patient characteristics	Number of patients	Dosing regimen	Other analgesic medication	Study design	Duration of active treatment (weeks)	Quality score	OPVS	50% or more pain relief	Other pain outcomes (detailed data extracted)
Wernicke et al. 2006	Age ≥18 yrs<br>PDN without any psychological disorder<br>Daily pain for ≥6 months<br>Baseline pain ≥4 (BPI 11 point Likert)	Mean age 61 (±11) yrs<br>M/F 204/130<br>Duration of neuropathy 4 yrs<br>White 78%<br>Hispanic 16%	334	(1) Dulox 60 mg/day. Fixed dose for 12 weeks, then 30 mg for 1 week, n=114<br>(2) Dulox 60 mg x1 daily for 3 days then x2 daily to 12 weeks then x1 daily for 1 week, n=112<br>(3) Placebo, n=108	Max 4 g paracetamol/day and 325g aspirin/day	R, DB, PC, parallel	13	R2, DB1, W1	13	(1) 43% (49/114)<br>(2) 53% (59/112)<br>(3) 27% (29/108)	Mean (SE) reduction 24hr APS<br>(1) 2.72 (0.22)<br>(2) 2.84 (0.23)<br>(3) 1.39 (0.23)
Arnold et al. 2004	Age ≥18 yrs<br>Primary fibromalgia with or without depression<br>Baseline pain ≥4 (FIQ 11 point scale)	Mean age 49 (±12) yrs<br>M/F 23/184<br>White 87%<br>Hispanic 5%<br>Current MDD 38%	207	(1) Dulox 20mg x1 daily for 5 days then 20mg x2 daily ≥3 days,then 40mg x2 daily for ≥2 days then 60mg x2 daily to 12 weeks, n=104<br>(2) Placebo, n=103	Max 2 g paracetamol/day and 325g aspirin/day	R, DB, PC, parallel	12	R2, DB2, W1	16	(1) 27.7% (29/104)<br>(2) 16.7% (17/103)	Mean reduction in pain FIQ (fibromyalgia impact questionnaire)<br>(1) 1.98 (0.3)<br>(2) 1.35 (0.29)
Raskin et al. 2005	Age ≥18yrs<br>PDN, without any psychological disorder<br>Pain present ≥6 months<br>Baseline pain ≥4 (BPI 11 point Likert)	Mean age 59 (±10) yrs<br>M162/F186<br>Caucasian 99.7%	348	(1) Dulox 60 mg x1 daily, n=116<br>(2) Dulox 60mg x1 daily for 3 days then 60mg x2 daily, n=116<br>(3) Placebo, n=116	Max 4 g paracetamol/day and 325g aspirin/day	R, DB, PC, parallel	12	R2, DB2, W1	16	(1) 50% (58/116)<br>(2) 39% (45/116)<br>(3) 30% (35/116)	Mean reduction (SE) 24hr APS<br>(1) 2.50 (0.18)<br>(2) 2.47 (0.18)<br>(3) 1.6 (0.18)
Goldstein 2005	Age ≥18yrs<br>PDN, without any psychological disorder<br>Pain present ≥6 months<br>Baseline pain ≥4 (BPI 11 point Likert)	Mean age 60 (±11) yrs<br>M281/F176<br>Caucasian 77%<br>Hispanic 11%	457	(1) Dulox 20 mg x1 daily, n=115<br>(2) Dulox 60mg x1 daily, n=114<br>(3) Dulox 40mg x 2daily for 1 week then 60mg x2 daily for 11 weeks, n=113<br>(4) Placebo, n=115	Max 4 g paracetamol/day<br>No other analgesics	R, DB, PC, parallel	12	R2, DB1, W1	13	(1) 41% (46/115)<br>(2) 49% (55/114)<br>(3) 52% (57/113)<br>(4) 26% (29/115)	Mean reduction (SE) 24hr APS<br>(1) 2.36 (0.21)<br>(2) 2.89 (0.22)<br>(3) 3.24 (0.23)<br>(4) 1.91 (0.22)
Arnold et al. 2005	Age ≥18yrs<br>Primary fibromyalgia with or without depression<br>Baseline pain ≥4 (BPI 11 point Likert)	Mean age 50 (±11) yrs<br>All F<br>Caucasian 90%<br>Hispanic 8%<br>Current MDD 26%	354	(1) Dulox 60 mg x1 daily, n=118<br>(2) Dulox 60mg x1 daily for 3 days then 60mg x2 daily, n=116<br>(3) Placebo, n=120	Max 2 g paracetamol/day and 325g aspirin/day	R, DB, PC, parallel	12	R1, DB1, W1	13	(1) 41% (48/116)<br>(2) 41% (47/114)<br>(3) 23% (27/118)	Mean reduction (SE) 24hr APS<br>(1) 2.39 (0.22)<br>(2) 2.40 (0.22)<br>(3) 1.16 (0.21)
Russell et al. 2008	Age ≥18yrs<br>ACR defined fibromyalgia with or without depression<br>Baseline pain ≥4 (BPI 11 point Likert)	Mean age 51 (±11) yrs<br>M 27/F 493<br>White 84.2%<br>Current MDD 24%	520	(1) Dulox 20 mg daily, n=115<br>(2) Dulox 30mg daily for 1 week, then 60mg daily, n=114<br>(3) Dulox 30mg daily for 1 week then 60mg daily for 1 week, then 120 mg daily, n=113<br>(4) Placebo, n=115	Max 2 g paracetamol/day and 325g aspirin/day	R, DB, parrallel	13	R2, DB1, W1	13	(1) 32% (20/79)<br>(2) 34% (51/150)<br>(3) 40% (59/147)<br>(4) 24% (34/144)	Mean reduction (SE) 24hr APS<br>(1) 1.92 (0.27)<br>(2) 1.99 (0.20)<br>(3) 2.31 (0.20)<br>(4) 1.39 (0.20)

Abbreviations: R=randomised; DB=double blind; W=withdrawals; PC=placebo controlled; BPI=brief pain inventory; AE=adverse event; PDN=painful diabetic neuropathy; Dulox=duloxetine; OPVS=Oxford Pain Validity Score; APS

Other outcomes	Ascertainment and reporting of adverse events	Specific adverse events	Withdrawals-total	Patients with any AE	Serious AE	Withdrawals-LoE	Withdrawals-AE
BPI average interference score<br>SF-McGill<br>HAMD total score<br>CGI severity	Adverse events, vital signs and lab tests evaluated<br>Ten most frequent specific adverse events reported	Nausea (1)32/114 (2)36/112 (3)7/108<br>Somnolence (1)9/114 (2)17/112 (3)1/108<br>Dizziness (1)18/114 (2)12/112 (3)6/108<br>Headache (1)12/114 (2)15/112 (3)7/108<br>Constipation (1)8/114 (2)21/112 (3)2/108<br>Insomnia, increased sweating, fatigue, nasopharyngitis also increased<br>Changes in lab tests and vital signs were small and not clinically relevant	(1) 29/114<br>(2) 34/112<br>(3) 23/108	(1) 102/114<br>(2) 96/112<br>(3) 79/108	(1) 5/114<br>(2) 2/112<br>(3) 5/108	(1) 1/114<br>(2) 3/112<br>(3) 5/108	(1) 17/114<br>(2) 20/112<br>(3) 8/108
BPI average interference score<br>CGI severity<br>PGI-I scale<br>FIQ total score<br>Beck depression inventory<br>Beck anxienty inventory	Adverse events, vital signs and lab tests evaluated	Insomnia, drymouth, constipation more frequent in dulox group than placebo group<br>Changes in lab tests (incl LFT) and vital signs were small and not clinically relevant	(1) 46/104<br>(2) 37/103	(1) 94/104<br>(2) 77/103	Not reported	(1) 9/104<br>(2) 13/103	(1) 18/104<br>(2) 11/103
BPI average interference score<br>MNSI (michigan neuropathy screening instrument)<br>HAMD total score<br>CGI severity	Vital signs and lab tests checked, and adverse evnets reviewed, at each visit.	Nausea,somnelence,hyperhidrosis and anorexia more frequent in dulox groups than placebo group. Vomiting and constipation more frequent in Dulox 60mg than placebo group.<br>Mean increases in lab tests (incl LFT, glucose, cholesterol) and vital signs were small, tansient and not clinicaly relevant.	(1) 15/116<br>(2) 21/116<br>(3) 16/116	(1) 71/116<br>(2) 73/116<br>(3) 57/116	(1) 4/116<br>(2) 2/116<br>(3) 4/116	Not reported	(1) 5/116<br>(2) 14/116<br>(3) 3/116<br><br>Most in first 4 weeks
BAI total (beck anxienty inventory)<br>Beck depression inventory<br>CGI severity	Spontaneously reported adverse events and vital signs recorded.<br>Specific events reported if significantly different from placebo	Nausea (1)16/115 (2)19/114 (3)31/113 (4)11/115<br>Somnolence (1)9/115 (2)23/114 (3)32/113 (4)9/115<br>Dizziness (1)7/115 (2)11/114 (3)26/113 (4)8/115<br>Constipation (1)6/115 (2)17/114 (3)12/113 (4)4/115<br>Dec apetite (1) 3/115 (2) 3/114 (3) 14/113 (4) 0/115<br>Drymouth, anorexia, weakness also sig different<br>Mean changes in lab tests (incl LFT) and vital [illegible]	(1) 24/115<br>(2) 28/114<br>(3) 33/113<br>(4) 28/115	Not reported	Total of 19 experienced serious adverse events	(1) 2/115<br>(2) 1/114<br>(3) 2/113<br>(4) 4/115	(1) 5/115<br>(2) 15/114<br>(3) 22/113<br>(4) 7/115
BPI average interference score<br>FIQ total score<br>CGI severity<br>PGI-I<br>HAMD<br>Sheehan Disability Scale<br>Medical Outcomes Study SF-36<br>QoL Depression Scale	Adverse events, vital signs and lab tests assessed<br>Specific events reported if significantly different from placebo	Nausea (1)52/118 (2)45/116 (3)16/120<br>Somnolence (1)6/118 (2) 14/116 (3) 5/120<br>Constipation (1)17/118 (2)20/116 (3)4/120<br>Dry mouth (1)25/118 (2)27/116 (3)10/120<br>Dec apetite (1) 6/118 (2) 13/116 (3) 2/120<br>Diarrhoea, anorexia, nasopharyngitis also increased<br>Changes in lab tests and vital signs were small and not clinically relevant	(1) 41/118<br>(2) 45/116<br>(3) 52/120	(1) 109/118<br>(2) 105/116<br>(3) 95/120	(1) 1/118<br>(2) 1/116<br>(3) 0/120	(1) 7/118<br>(2) 4/116<br>(3) 18/120<br>NB 13 patients in placebo group withdrew consent (vs 1 and 4 in dulox 60 and 120mg groups)	(1) 25/118<br>(2) 27/116<br>(3) 14/120
PGI-I (patient global impression of improvement)<br>FIQ total score<br>CGI severity<br>Multidimensional Fatigue Inventory<br>HAMD<br>Sheehan Disability Scale<br>Medical Outcomes Study SF-36<br>Euro-QoL Questionnaire-5D	Spontaneously reported adverse events, vital signs and weight recorded.	No separate data for first 13 weeks	(1) 30/79<br>(2) 53/150<br>(3) 52/147<br>(4) 84/144	Not reported	No separate data for first 13 weeks	(1) 8/79<br>(2) 11/150<br>(3) 6/147<br>(4) 14/144	(1) 8/79<br>(2) 22/150<br>(3) 32/147<br>(4) 17/144

[illegible]=average pain score; HAMD=Hamilton depression score; CGI=clinical global impression; PGI-I=patient global improvement; FIQ=fibromyalgia impact questionnaire
